# The Status of Nutritional Management Guidelines for Head and Neck Cancer Patients

**DOI:** 10.7759/cureus.11309

**Published:** 2020-11-03

**Authors:** Patricia Curtin, Aelia Akbar, Holly Kramer, Aqsa Iqbal, Talar Markossian

**Affiliations:** 1 Medicine, Loyola University Medical Center, Chicago, USA; 2 Internal Medicine, Loyola University Medical Center, Chicago, USA; 3 Nephrology, Loyola University Medical Center, Chicago, USA; 4 Cardiology, University of Illinois at Chicago, Chicago, USA; 5 Epidemiology and Public Health, Loyola University Medical Center, Chicago, USA

**Keywords:** oncology, nutritional, guidelines, head and neck cancer, management

## Abstract

Introduction

Head and neck cancer (HNC) is the seventh leading cause of cancer worldwide. Approximately 35%-60% patients with HNC are malnourished from the disease onset, malnutrition being associated with worsened health outcomes among these patients. This study aimed to review and synthesize existing guidelines regarding nutritional interventions in HNC patients and assess providers’ knowledge, opinions, and practice of guidelines for the nutritional management of HNC patients.

Methods

This is a multimethod study that includes a systematic review of guidelines for nutritional intervention in HNC patients and a providers’ survey regarding their knowledge and opinions regarding nutrition therapy guidelines for HNC patients.

Results

Our review yielded seven guidelines. Of the seven guidelines reviewed, all were specific to cancer patients, however, only three were specific for HNC patients. Three of the guidelines recommended using a nutritional screening tool, however, only two mentioned a specific screening tool. Out of 193 surveys included in our analysis, the highest percentage of respondents were physicians (52.4%), followed by registered nurses (33.5%). The majority of respondent (77.5%) worked in a hospital-based practice, while 18.8% worked in clinic-based practice. A large proportion (46.6%) of respondents were not aware of nutritional guidelines for HNC patients; with 23.6% not aware of any, and 23.0% aware of their existence but not aware of their content. The majority (81.5%) of respondents said that a more detailed guideline should be available for HNC patient with regards to nutrition.

Conclusion

Nutritional deficiencies in HNC patients continue to cause significant complications in treatment and recovery. Existing practice guidelines are limited and lack specific recommendations. A universal standard of care with regard to addressing nutrition in HNC patients is needed to improve healthcare outcomes among NHC patients.

## Introduction

Head and neck cancer (HNC) is the seventh leading cause of cancer worldwide [1]. It encompasses malignancies arising from mucosal surfaces of the oral cavity, pharynx, larynx, and paranasal sinuses, as well as cancers of the major and minor salivary glands [2]. According to the National Cancer Institute’s (NCI’s) Surveillance, Epidemiology, and End Results (SEER) more than 65,000 new cases of HNC are projected in 2020 with 14,500 deaths attributable to the disease [1]. The causative risk factors for HNC are tobacco use [3], alcohol use, and human papillomavirus (HPV) infection [2]. Studies have shown that smokers consume less fruits and vegetables than non-smokers [4], in turn, low carotenoid intake and other protective components of fruits and vegetables is associated with increased risk of HNC [5]. Approximately 35%-60% patients with HNC are malnourished from the disease onset [6]. In addition, tumor location and side effects of multimodal therapy further places HNC patients at increased risk of malnutrition [7,8]. During radiation therapy, 44%-88% of HNC patients are malnourished [8-10]. Malnutrition can be defined as an unintentional weight loss of greater than 5% in three months or 10% in six months [11] or body mass index (BMI) of less than 20 kg/m^2^ [11,12]. Albumin deficiency with albumin level less than 35 g/L in an acutely ill patient also suggests malnutrition [13-16]. Malnutrition causes decreased response to therapy, decreased immunocompetency, increased risk of infections, increased post-operative complication rates, and decreased survival rates [14,17,18]. Therefore, a delayed diagnosis of malnutrition in HNC patient may result in delayed nutritional intervention causing increased mortality in these patients. Nutritional interventions such as weight monitoring and nutritional counseling have been shown to improve health outcomes in HNC patients by decreasing weight loss, improving quality of life, and physical function [19]. It is imperative that HNC patients are screened in a timely manner for malnutrition and referred for nutrition management [20].

There are some screening methods that are available for the early diagnoses of malnutrition among HNC patients [12,20]. Skipper et al. reviewed eleven different screening tools [21] and Cascio et al. reviewed five different nutritional screening tools [22]. Both studies compared nutrition screening tools for reliability and validity, and found significant differences. These studies showed that there should be a simple, easy to use and universal screening tool for malnutrition which can be used as standard across healthcare. There are several guidelines available for nutritional management in this complex patient population. However, these guidelines are not consistent in terms of screening methods, timeliness of referral, or management.

This study had two main objectives; (1) to conduct a systematic review and analysis of existing guidelines regarding nutritional interventions in HNC patients and (2) to assess providers’ knowledge and practice of guidelines for the nutritional management of HNC patients. A providers’ survey also queried whether more comprehensive, detailed guidelines with regard to nutritional treatment in HNC patients are needed.

## Materials and methods

This is a multimethod study that includes a systematic review of guidelines for nutritional intervention in HNC patients and a providers’ survey regarding their knowledge and opinions regarding nutrition therapy guidelines for HNC patients.

Guidelines review and analysis

Data Sources

A systematic review was conducted using ClinicalKey, The Cumulative Index to Nursing and Allied Health Literature (CINAHL), and the National Guideline Clearinghouse (NGC), which are clinical guideline databases. The search string [head AND neck AND cancer AND nutrition] was used. The term ‘guideline’ was not included, since these were all clinical guidelines databases. A similar search was also performed in PubMed by adding the term guideline, the search string included [head AND neck AND cancer AND nutrition AND guideline]. We analyzed the guidelines and included them in our review if they addressed nutritional intervention in HNC patients. Hits that were not related to HNC were excluded. Guidelines from national and international organizations were included. 

Our review yielded seven guidelines. The following guidelines were found using each search database; (1) ClinicalKey: National Comprehensive Cancer Network Guidelines (NCCN), National Institute for Health and Care Excellence (NICE), and Academy of Nutrition and Dietetics (AND). (2) CINAHL: Evidence-Based Care Sheet: Squamous Cell Carcinoma: Laryngeal and Hypopharyngeal Cancer, and Evidence-Based Care Sheet: Squamous Cell Carcinoma: Nasopharyngeal Cancer. (3) NGC: Academy of Nutrition and Dietetics (AND). (4) PubMed: American Society for Parenteral and Enteral Nutrition (ASPEN), European Society for Clinical Nutrition and Metabolism (ESPEN), National Comprehensive Cancer Network (NCCN), and Academy of Nutrition and Dietetics (AND).

Data Extraction and Synthesis

We reviewed each guideline and extracted the following information: (1) If the guideline was specifically for HNC patients, (2) the target audience for the guideline (type of practitioner), (3) if it included recommendation for referral to registered dietitian, (4) what was the time frame for referral to registered dietitian, (5) If it included recommendation for a nutritional screening tool and which tool, and (6) if it includes recommendations regarding tube feeding (Table 1). 

**Table 1:** Comparison of clinical guidelines for managing nutrition in head and neck cancer (HNC) patients *All guidelines were specific to cancer patients *None had specific guidelines for elderly patients *None specified whether they were in-patient or out-patient care.

Guidelines	Are guidelines specific for HNC patients	Number of pages	Target population (type of practitioner)	Recommends referral to registered dietician	Time frame for referral to registered dietician	Recommends a nutritional screening tool	Nutritional screening tool	Gives recommendations regarding tube feeding
Nutritional Comprehensive Cancer Network (NCCN) guidelines (version 1.2016): Head and neck cancers “Principles of Nutrition: Management and supportive care”	Yes	2	Not Specified	Yes	With the initiation of treatment	Yes	Use of screening tool is recommended. However, a specific malnutrition screening tool is not recommended	yes
American society for parenteral and enteral nutrition (ASPEN) Clinical guidelines: Nutritional support therapy during adult anticancer treatment and in hematopoietic stem cell transplantation	No	1	Not specified	No	N/A	No	N/A	No
Academy of nutrition and dietetics (AND) Oncology 2013 Evidence Based Nutrition Practice Guidelines	No	15	Registered Dieticians, Advanced Practice Nurses, Healthcare Providers, Health Plans, Hospitals, Managed Care Organizations, Nurses, Physician Assistants, Students.	Yes	All adults patients should be screened for malnutrition risk on entry into oncology services…if an adult oncology patient is identified at screening to be at risk for malnutrition, the patient should be referred to a registered dietician.	Yes	Patient Generated Subjective Global Assessment (PG_SGA). Malnutrition Universal Screening Tool (MUST). Malnutrition Screening Tool (MST). Malnutrition Screening Tool for Cancer Patients (MSTC)	No
American Head and Neck Society (AHNS)	AHNS are no longer available due to their age. They recommend using NCCN guidelines.							
CINAHL Nursing Journal Database (Evidence Based Care Sheet)	Yes	4 (1 line regarding nutrition)	Nurses	Yes	None	No	No	No
The European Society for Clinical Nutrition and Metabolism (ESPEN)	No	38	Clinical oncologists, Healthcare providers involved in supportive care of cancer patients and cancer survivors e.g. medical specialists involved in cancer treatment, family physicians, pharmacists, nurses, dieticians, nutritionists, and exercise physiologists, as well as medical leaders and administrators or oncological institutes.	Yes	Not clearly specified	Yes	Use of a nutrition screening tool is recommended but multiple screening tools are given as examples: MUST, MST, Mini Nutritional Assessment Short Form Revised.	Yes
National Institute for Health and Care Excellence (NICE) (UK)	Yes	3	Service Providers Healthcare Providers Commissioners.	No	N/A	No	N/A	Recommends nutritional assessment at diagnosis, including the need for tube feeding.

Provider survey of nutritional interventions in HNC patients 

Target Population and Study Design

The target population consisted of healthcare providers within the United States who worked with HNC patients. A cross-sectional survey was distributed to the members of the Oncology Nursing Society (ONS) from March 5 to April 23, 2018. ONS consisted of Registered Nurses, Nurse Practitioners, Registered Dieticians and other healthcare professionals. The survey was also distributed among members of the American Head and Neck Society (AHNS) which consisted of physicians, Nurse Practitioners, Physician Assistants, and other healthcare professionals from February to December 2019. The emails of the members were directly accessed from ONS and AHNS.

The Survey Instrument

The survey instrument consisted of 32 questions. It was administered online via Survey Monkey. The questions can be found in the attached Appendix. The questions focused on three categories: providers’ knowledge and practice, providers’ opinions, and providers’ demographics information. The first part of the survey, questions 2 to 19 collected information regarding providers’ knowledge and use of practice guidelines regarding nutrition in HNC patients. The second part of the survey, from questions 20 to 26 collected providers’ opinions on current guidelines and their efficacy, whether healthcare providers perceive there to be a need for a universal standard of care with regard to screening for malnutrition in HNC patients, and whether healthcare providers perceive there to be a need for more detailed, comprehensive treatment guidelines with regard to HNC patients. Third part of the survey, questions 27 to 32, included questions on providers’ information with regards to their age, sex, institution, and current position as a healthcare provider. 

The participants were not asked to provide any identifying information, as the survey was anonymous.

Study procedures

The ONS and AHNS keep databases of email addresses of its members. An email invitation with a web link to the survey was emailed to members of the ONS and AHNS. Participants were sent one additional email several days later as a reminder. In the survey, the respondents were asked if they saw head and neck cancer patients in their practice. If the response was “Yes”, they progressed to the next question. If the response was “No”, a disqualification message thanked the respondent for their time. Out of the total 196 responses from ONS, 24 were automatically disqualified, and out of 141 responses from AHNS, 1 was disqualified due to the fact that the respondent did not see head and neck cancer patients in their practice. Thus, total of 312 respondents from ONS and AHNS saw head and neck patients. Out of these, 119 did not complete the questionnaire and their surveys were excluded from the analysis. In summary, we received a total of 337 surveys, we excluded if the participant did not see HNC patients (n=25) and did not complete the questionnaire (n=119). Finally, a total of 193 surveys were included in the analysis. 

Ethical statement

Solutions institutional review board (IRB) ruled that the study is exempt as the survey is anonymous.

Statistical analysis

One of the authors reviewed all the guidelines using the data extraction tool and summarized the results in a descriptive (Table 1). Two authors met weekly to review the results and address questions related to the extraction process. Results from the survey were presented in descriptive tables using univariate statistics. Participant demographics are described in Table 2 and participant responses are summarized in Tables 3-4. All analysis of survey data were conducted in Stata version 14.1. 

**Table 2:** Characteristics of the study participants who completed the survey questionnaire on Nutritional Intervention in Head and Cancer Patients (n=193) * The total number of responses may not add to 193 in all questions because of missing data in some responses.

Characteristics		Number	Percentage (%)
Type of Provider	Registered Nurses	64	33.5
	Nurse Practitioners	5	2.6
	Physician Assistants	2	1.0
	Physicians	100	52.4
	Registered Dietitians	3	1.6
	Other	17	8.9
Type of Institution	Hospital Based Practice	148	77.5
	Clinic Based Practice	36	18.8
	Others	7	3.7
Gender	Males	78	41.0
	Females	112	58.9
Age (years)	24 and younger	3	1.6
	25-44	90	47.4
	45-74	94	49.5
	75 and older	3	1.6
Years in Practice	Up to 9 years	73	38.3
	10-19	42	22
	20 or more	76	39.8
Location	Northeastern United States	31	16.2
	Southeastern United States	35	18.3
	Southwestern United States	24	12.6
	Midwestern United States	54	28.3
	Intermountain Region	13	6.8
	Pacific Northwest Region	12	6.3
	Outside of the United States	22	11.5

**Table 3:** Responses to questions on provider knowledge and use of guidelines * The total number of responses may not add to 191 in all questions because of missing data in some responses. ** the total number of responses in Question 2a to 2f may not add to 101 because of missing data.

Provider knowledge and Use of Guidelines	Number	Percent (%)
1. Which of the following best describes your knowledge of practice guidelines regarding head and neck cancer (HNC) patients?		
I am not aware of any guidelines regarding nutrition in head and neck cancer patients.	45	23.6
I am aware of their existence, but I am not aware of their contents.	44	23.0
I am aware of only my institution's practice guidelines.	45	23.6
I am aware of practice guidelines designed by a professional organization.	27	14.1
I am aware of multiple practice guidelines regarding nutrition in head and neck cancer patients.	30	15.7
2. Does your institution have guidelines with regard to addressing nutrition in head and neck cancer patients?		
Yes	102	52.8
No	91	47.1
Among those who answered ‘yes’ in question #2 (2.a. – 2.f.) (n=102)		
2.a. Are your institution's guidelines institution-designed, or are they adopted from the practice guidelines of a professional organization?		
Institution-designed	45	44.1
Adopted from a professional organization	38	37.2
N/A	19	18.6
2.b. Do you know where to find your institution's guidelines with regard to addressing nutrition in head and neck cancer patients?		
Yes	67	67.0
No	32	32.0
N/A	1	1.0
2.c. When was the last time you reviewed your institution's guidelines with regard to addressing nutrition in head and neck cancer patients?		
Within the last week	7	6.9
Within the last month	14	13.9
Within the last year	38	37.6
More than a year ago	18	17.8
Never	24	23.8
2.d. Do you regularly use your institution's guidelines with regard to addressing nutrition in head and neck cancer patients?		
Yes	60	60.0
No	27	27.0
N/A	13	13.0
2.e. How would you rate the effectiveness of your institution's guidelines with regards to addressing nutrition in head and neck cancer patients? (Do you feel that nutritional risks in HNC cancer patients are being addressed in a timely manner?		
Very effective	28	27.7
Effective	39	38.6
Adequate	16	15.8
Could use improvement	13	12.9
Ineffective	1	0.9
N/A	4	3.9
2.f. How would you rate the thoroughness of your institution's guidelines with regard to addressing nutrition in head and neck cancer patients? (Do you feel that the guidelines address all of the issues they should?)		
Very thorough	34	34.0
Somewhat thorough	26	26.0
Adequate	15	15.0
Could use improvement	15	15.0
Not thorough	4	4.0
N/A	6	6.0
3. Do you have any nutritional handouts/pamphlets that you give out to new head and neck cancer patients?		
Yes	92	48.9
No	96	51.1
4. How helpful do you find the NCCN Guidelines regarding nutrition in head and neck cancer patients?		
Very helpful	36	19.6
Somewhat helpful	45	24.5
Not very helpful	5	2.7
Not helpful at all	1	0.5
No opinion	97	52.7
5. Do you feel that more detailed guidelines with regard to nutrition in head and neck cancer patients would improve patient care?		
Yes	150	81.5
No	34	18.5

**Table 4:** Responses to questions on nutritional screening in head and neck cancer patients *The total number of responses may not add to 191 in all questions because of missing data in some responses.

Nutritional Screening in Head and Neck Cancer Patients		Number	Percent (%)
Do you (personally) screen head and neck cancer patients for malnutrition?			
Yes		114	59.7
No		77	40.3
Does your institution have a designated healthcare professional who screens for malnutrition in head and neck cancer patients?			
Yes		130	68.1
No		61	31.9
If you (personally) screen for malnutrition in head and neck cancer patients, do you use a Nutrition Screening Tool?			
Yes		41	25.5
No		120	74.5
If you do screen for malnutrition, which screening tool do you use?			
Malnutrition Screening Tool (MST)		22	22.0
Mini Nutrition Assessment-Short Form (MNA-SF)		5	5.0
Malnutrition Universal Screening Tool (MUST)		5	5.0
Nutritional Risk Screening 2002 (NRS)		12	12.0
Other		56	56.0
Does your institution have a protocol in place for referring head and neck cancer patients to see a registered dietitian?			
Yes		155	81.6
No		35	18.4
If your response to the previous question was "yes", when do you refer the patient to a registered dietitian?			
At or as soon after diagnosis as possible		92	53.8
At start of treatment		30	17.5
At a designated point during treatment		10	5.8
Only when the patient is diagnosed with malnutrition		23	13.4
Never		5	2.9
Other		11	6.4
How often are head and neck cancer patients seen by registered dietitians during treatment?			
Never		1	0.5
One time		6	3.2
Weekly		44	23.7
Monthly		6	3.2
As Needed		113	60.7
At another designated interval		16	8.6
How compliant do you feel patients are with regard to seeing registered dietitians once referred?			
Always compliant		37	19.7
Somewhat compliant		128	68.1
Rarely compliant		20	10.6
Not compliant		3	1.6
Do you think a universal standard of care with regard to screening for malnutrition in head and neck cancer patients would improve healthcare outcomes?			
Yes		177	93.2
No		13	6.8

## Results

Guideline review and analysis

Our review yielded seven guidelines (as seen in Table 1), but one guideline (AHNS) is considered outdated and no longer available. The NCCN, CINAHL and NICE provide specific guidelines for HNC patients while ASPEN, AND, and ESPEN guidelines are not specific for HNC patients. The NCCN and ASPEN have not specified the target population but CINAHL are directed towards nurses specifically. The AND, ESPEN and NICE state the guidelines are targeted at healthcare providers which include, Registered dieticians, nurses, Physician assistants, and others. Four of the guidelines (NCCN, AND, CINAHL, ESPEN) recommend referring the HNC patients to a registered dietician. NCCN recommends referring to dietician with initiation of treatment while AND recommends screening of patients for malnutrition and then referring to dietician if they are found malnourished. Only NCCN, AND, ESPEN, recommend a nutritional screening tool but do not specify any single screening tool. The tube feeding is recommended by the NCCN, ESPEN, and NICE. 

Provider survey of nutritional interventions in HNC patients

Out of 4246 invitations to member of ONS, 196 (4.6%) responses were received, and out of 1450 invitations to AHNS, 141 (9.7%) responses were received, with total response of 337 out of the total of 5696 invitations (5.9%). Out of 193 surveys included in our analysis, the highest percentage of respondents were physicians (52.4%), followed by registered nurses (33.5%). The majority of respondent (77.5%) worked in a hospital-based practice, while 18.8% worked in clinic-based practice. Higher percentage of respondents were female (58.9%). Of the 193 respondents, 1.6% were age 24 years or younger, 47.4% were between the ages of 25-44 years, 49.5% were between the ages of 45-74 years and 1.6% were age 75 years and older. While 38.3% of the respondents had been practicing for up to nine years, 22% had been practicing between 10 and 19 years, and 39.8% had been practicing 20 years or more. The scope of this study included all areas of the United States, with the largest percentage of respondents being from the Midwest (28.3%) (Table 2).

The first part of survey was focused on providers’ knowledge about nutritional guidelines in HNC patients (Table 3). A large proportion (46.6%) of respondents were not aware of nutritional guidelines for HNC patients; with 23.6% not aware of any, and 23.0% aware of their existence but not aware of their content. Out of those who were aware of these guidelines (53.4%); 23.6% were aware of only their institution’s guidelines, 14.1% were aware of some professional institution’s guidelines, and only 15.7% were aware of multiple practice guidelines for nutrition in HNC patients. In addition, 52.8% (102 out of 193) respondents reported that their institution had practice guidelines for nutritional management in HNC patients. Out of these 102 respondents, 44.1% reported that the guidelines were designed by their institution and 37.2% reported that they were adopted from a professional organization. Additionally, only 67% of them were aware of where to find their institution’s guidelines regarding nutrition in HNC patients, with 23.8% who had never reviewed them. Out of 102 respondents who had these guidelines at their institution, 60% regularly used them to address nutrition management in HNC patients. Most (82.1%) of the respondents noted the institution’s guidelines are either effective or adequate in nutritional management of HNC patients, and 75% noted that their institution’s guidelines were either thorough or adequate. Only 13.8% either said that their institutions guidelines were not effective or needs improvement.

Approximately half of our total respondents (48.9%) provided their HNC patients with informational handouts regarding nutrition. In addition, 44.1% of respondents found NCCN guidelines helpful. Finally, majority (81.5%) respondents said that a more detailed guidelines should be available for HNC patient with regards to nutrition. 

The second part of survey was focused on nutritional screening in HNC patients (Table 4). Most of the providers (59.7%) personally screened their patients for malnutrition and 68.1% of total also had a designated professional who screened patients for malnutrition. Only 25.5% used a nutrition screening tool; 22% used MST, 5% used MNA-SF, 5% used MUST, 12% used NRS, and the remining 56% used other tools. Majority (81.6%) of respondents had a protocol at their institutions to refer the HNC patient to a registered dietician; with 53.8% to refer as soon as the diagnosis of HNC is made and 2.9% who don’t require referring the patient to registered dietician. While 23.3% required patients to be referred to dietician at the start or sometime during treatment, 13.4% required to refer them only after a diagnosis of malnutrition was made. All of the respondents excluding one reported that patients are seen by registered dietician during treatment, and 23.7% said that they are seen weekly, while 60.7% said they are seen as needed. Majority of the respondents also answered that patients are compliant in seeing the registered dietician; 19.7% noted they are always compliant and 68.1% noted that they are somewhat compliant. Almost all (93.2%) of respondents noted that a universal and standard malnutrition screening tool can improve healthcare outcomes for HNC patients.

## Discussion

Malnutrition in cancer patients significantly decreases response to therapy and increases the risk of complications and toxicities [23]. Malnutrition leads to impaired immune response, reduced muscle strength, increased fatigue, impaired wound healing, impaired psycho-social function, and reduced quality of life [14]. These factors can lead to increased rates of hospital admissions, readmissions, length of stay, and significant healthcare costs [17,18]. Furthermore, the nutritional status of head and neck cancer patients is often compromised before treatment even begins due to an altered ability to chew, swallow, or taste, resulting from the physical location of the tumors [4-10,24,25]. 

We found seven nutritional guidelines for cancer patients and three NCCN, CINAHL, and NICE were specific for HNC patients. Three of the guidelines recommended using a nutritional screening tool, however, only two (AND, and ESPEN) mentioned a specific screening tool. Five of the seven guidelines recommend referral of the patients to a registered dietitian, however, only two specified a time frame for referring to a dietitian. The NCCN recommends referring to dietician with the start of treatment, while AND recommend screening all cancer patients for malnutrition and referring to dietician if found to be at risk for malnutrition. There is a lack of knowledge among healthcare providers who see HNC patients regarding these guidelines and providers are often not aware of their own institution’s guidelines. While there are many malnutrition screening tools available and some guidelines such as NCCN, ESPEN and AND recommend using them, majority of providers do not use them. Additionally, inconsistencies are found in timeframes for referring to registered dieticians amongst healthcare providers. Finally, majority of providers who see HNC patients recommended a detailed and universal guideline for nutritional intervention in HNC patients which can improve healthcare outcomes for HNC patients. A universal screening tool would allow non-registered dietitian healthcare professionals to screen for malnutrition at the time of diagnosis allowing for quicker interventions.

Strengths and limitations

A major strength of our study was the inclusion of healthcare providers including physicians, dieticians, nurses, and others across all regions of the United States who saw HNC patients to obtain a more generalized assessment. However, the number of providers that responded to survey was small. There is also the potential for self-selection bias, in that providers and patients with a particular interest in nutrition may have been more likely to respond to the survey, and for non-response bias. Additionally, there is the potential for social desirability bias, in that participants may have responded in a manner that they believed would be viewed favorably by the study team, although this risk may have been reduced by administering the surveys online and assuring respondents of their anonymity. 

## Conclusions

Nutritional deficiencies in HNC patients continue to cause significant complications in treatment and recovery. While this issue is well documented, it has not been effectively addressed. Existing practice guidelines are limited and lack specific recommendations. A universal standard of care with regard to addressing nutrition in head and neck cancer patients is needed to improve healthcare outcomes among NHC patients. 

## Appendices

Nutrition in HNC patients questionnaire

1. Do you treat patients with head and neck cancers?

·       Yes 

·       No

If your answer to question 1 is yes, please continue with the survey. If your answer is no, there is no need to continue, but your time is greatly appreciated.

2. Which of the following best describes your knowledge of practice guidelines regarding nutrition in head and neck cancer (HNC) patients?

·       I am not aware of any guidelines regarding nutrition in head and neck cancer patients.

·       I am aware of their existence, but I am not aware of their contents.

·       I am aware of only my institution's practice guidelines.

·       I am aware of practice guidelines designed by a professional organization.

·       I am aware of multiple practice guidelines regarding nutrition in head and neck cancer patients.

3. Does your institution have guidelines with regard to addressing nutrition in head and neck cancer patients?

·       Yes

·       No

4. Are your institution’s guidelines institution-designed, or are they adopted from the practice guidelines of a

·       professional organization?

·       Institution-designed

·       Adopted from a professional organization

·       N/A

5. If your answer to the previous question is "adopted from the practice guidelines of a professional organization", which organization's guidelines do they use?

6. Do you know where to find your institution’s guidelines with regard to addressing nutrition in head and neck cancer patients?

·       Yes

·       No

·       N/A

7. When was the last time you reviewed your institution’s guidelines with regard to addressing nutrition in head and neck cancer patients?

·       Within the last week

·       Within the last month

·       Within the last year

·       More than a year ago

·       Never

·       N/A

8. Are you familiar with the section of the National Comprehensive Cancer Network (NCCN) Guidelines that were published at the beginning of year 2016 (Version 1.2016): Head and Neck Cancers titled “Principles of Nutrition: Management and Supportive Care”?

·       Yes

·       No

9. Are you familiar with the recent additions to the section of the NCCN Guidelines that were published at the beginning of year 2016 (Version 1.2016): Head and Neck Cancers titled “Principles of Nutrition: Management and Supportive Care” regarding alternative routes for nutrition?

·       Yes

·       No

10. If you were to name one difference between your institution’s guidelines and the NCCN guidelines, what would it be?

11. Do you regularly use your institution’s guidelines with regard to addressing nutrition in head and neck cancer patients?

·       Yes

·       No

·       N/A

12. Do you (personally) screen head and neck cancer patients for malnutrition?

·       Yes

·       No

13. Does your institution have a designated healthcare professional who screens for malnutrition in head and neck cancer patients?

·       Yes

·       No

14. If you (personally) screen for malnutrition in head and neck cancer patients, do you use a Nutrition Screening Tool?

·       Yes

·       No

15. If you do screen for malnutrition, which screening tool do you use?

·       Malnutrition Screening Tool (MST)

·       Mini nutrition assessment-short form (MNA-SF)

·       Malnutrition Universal Screening Tool (MUST)

·       Nutritional Risk Screening 2002 (NRS)

·       Other (please specify)

16. Does your institution have a protocol in place for referring head and neck cancer patients to see a registered dietitian?

·       Yes

·       No

17. If your response to the previous question was “yes”, when do you refer the patient to a registered dietitian?

·       At or as soon after HNC diagnosis as possible

·       At start of treatment

·       At a designated point during treatment

·       Only when the patient is diagnosed with malnutrition

·       Never

·       Other (please specify)

18. How often are head and neck cancer patients seen by registered dietitians during treatment?

·       Never

·       One time

·       Weekly

·       Monthly

·       As needed

·       At another designated interval (please specify)

19. How compliant do you feel patients are with regard to seeing registered dietitians once referred?

·       Always compliant

·       Somewhat compliant

·       Rarely compliant

·       Not compliant

20. How would you rate the effectiveness of your institution’s guidelines with regard to addressing nutrition in

head and neck cancer patients? (Do you feel that nutritional risks in HNC patients are addressed in a timely manner?)

·       Very effective

·       Effective

·       Adequate

·       Could use improvement

·       Ineffective

·       Very ineffective

·       N/A

21. How would you rate the thoroughness of your institution’s guidelines with regard to addressing nutrition in head and neck cancer patients? (Do you feel that the guidelines address all of the issues they should?)

·       Very thorough

·       Somewhat thorough

·       Adequate

·       Could use improvement

·       Not thorough

·       N/A

22. Do you have any nutritional handouts/pamphlets that you give out to new head and neck cancer patients?

·       Yes

·       No

23. Do you think a universal standard of care with regard to screening for malnutrition in head and neck cancer patients would improve healthcare outcomes?

·       Yes

·       No

24. How helpful do you find the NCCN Guidelines regarding nutrition in head and neck cancer patients?

·       Very helpful

·       Somewhat helpful

·       No opinion

·       Not very helpful

·       Not helpful at all

25. Do you have any specific recommendations for improving practice guidelines for nutrition in head and neck cancer patients? If you would like to share them, please do so below.

26. Do you feel that more detailed guidelines with regard to nutrition in head and neck cancer patients would improve patient care?

·       Yes

·       No

27. Your current position:

·       Certified Nursing Assistant

·       Licensed Practical Nurse

·       Registered Nurse

·       Nurse Practitioner

·       Physician Assistant

·       Physician

·       Registered Dietitian

·       Other (please specify)

28. Type of institution:

·       Hospital-based practice

·       Private practice

·       Health center

·       Academic medical center

·       Military

·       Other (please specify)

29. Age:

·       24 or younger

·       25-34

·       35-44

·       45-54

·       55-64

·       65-74

·       74 or older

30. Gender:

·       Male

·       Female

31. Years in practice:

·       Less than 5

·       5-9

·       10-14

·       15-19

·       20 or more

32. Where is your institution located?

·       Northeastern United States

·       Southeastern United States

·       Midwestern United States

·       Intermountain Region of the United States

·       Pacific Northwestern United States

·       Southwestern United States

·       Alaska

·       Hawaii

·       Outside of the United States/Other (please specify)
